# Factors associated with access and use of PPE during COVID-19: A cross-sectional study of Italian physicians

**DOI:** 10.1371/journal.pone.0239024

**Published:** 2020-10-12

**Authors:** Elena Savoia, Giorgia Argentini, Davide Gori, Elena Neri, Rachael Piltch-Loeb, Maria Pia Fantini

**Affiliations:** 1 Emergency Preparedness Research, Evaluation, & Practice (EPREP) Program, Division of Policy Translation & Leadership Development, Harvard T. H. Chan School of Public Health, Boston, MA, United States of America; 2 IRCCS Burlo Garofolo, Trieste, Italy; 3 Department of Biomedical and Neuromotor Sciences (DIBINEM), University of Bologna, Bologna, Italy; 4 Azienda Sanitaria Giuliano Isontina, Trieste, Italy; Chinese Academy of Medical Sciences and Peking Union Medical College, CHINA

## Abstract

**Objectives:**

During the course of the Novel Coronavirus (SARS-CoV-2) pandemic, Italy has reported one of the highest number of infections. Nearly ten percent of reported coronavirus infections in Italy occurred in healthcare workers. This study aimed to understand physicians’ access to personal protective equipment (PPE) and to information about their use, risk perception and strategies adopted to prevent contracting the infection.

**Methods:**

We undertook a cross-sectional, online self-reported survey implemented between March 31 and April 5 2020 of Italian physicians.

**Results:**

Responses were received from 516 physicians, only 13% of which reported to have access to PPE every time they need them. Approximately half of the physicians reported that the information received about the use of PPE was either clear (47%) or complete (54%). Risk perception about contracting the infection was influenced by receiving adequate information on the use of PPE. Access to adequate information on the use of PPE was associated with better ability to perform donning and doffing procedures [OR = 2.2 95% C.I. 1.7–2.8] and reduced perception of risk [OR = 0.5, 95% C.I. 0.4–0.6].

**Conclusions:**

Results from this rapid survey indicate that while ramping up supplies on PPE for healthcare workers is certainly of mandatory importance, adequate training and clear instructions are just as important.

## Introduction

Globally, as the Novel Coronavirus (SARS-CoV-2) pandemic has evolved there has been a shortage of personal protective equipment (PPE) available to the healthcare workforce [1, 2]. As the World Health Organization has warned since the beginning of March, disruption to the global supply of PPE, has left frontline healthcare workers ill-equipped to care for their patients [2, 3]. Since the start of the epidemic, guidance on the usage of such equipment has continued to evolve, and has emphasized conservation of resources rather than optimizing protection of workers [4].

The coronavirus pandemic has taken a dramatic toll worldwide and especially in Italy. As of the beginning of April, Italy has reported one of the highest number of infections and the highest number of deaths of any European country [5]. Media reports from across Italy have shone a light on the burden that the coronavirus is placing on health workers. Nearly ten percent of reported coronavirus infections in Italy occurred in healthcare workers [6]. As of April 15, 155,467 cases and 19,508 deaths attributed to COVID-19 were confirmed in the country, and the number of healthcare workers infected and those that lost their life due to COVID-19 was 16,650 and 149 respectively [6, 7]. Many of these infections are likely due to occupational hazard; workers becoming infected while caring for patients suggesting the shortage or inappropriate use of PPE may be at the root of part of these infections.

The use of PPE has been identified as one of the biggest physical and psychological challenges experienced by physicians while responding to COVID-19 [8]. For example, physical burdens related to PPE include repeated donning and doffing of equipment and extended hours wearing uncomfortable masks and respirators, while psychological burdens include challenges communicating with peers and patients when wearing PPE and operating under changed practice standards. Because of PPE shortages, healthcare workers, who may have been trained on how to don and doff PPE to maximize protection from infection, have had to make ad hoc adjustments on what piece of equipment to use and when, that are not reflected in any training they have received. The additional burdens created by a shortage whereby processes for using PPE are continuously changing, has not been explored.

The Italian Healthcare System is regionally based and organized at the national, regional, and local levels, with each region having the autonomy of managing the delivery of the healthcare services based on local needs [9]. The Italian National Healthcare System certifies healthcare workers and requires them to complete continuing education credits, while quality and standards of care are set by the regions and hospitals. Training procedures for the healthcare workforce are also left to the regions and local hospitals, specifically regarding the use and management of PPE. Such differences are expected given local needs and hospital settings differ by localities, however such differences may also have caused inconsistencies and confusion on the appropriate use of PPE in a rapidly evolving situation such as the COVID-19 outbreak. Currently, there is lack of literature on how the healthcare workforce in Italy has adapted during the Novel Coronavirus pandemic in the use of equipment. This study aimed to understand physicians’ access to PPE, reception of information about their use, ability to perform donning and doffing procedures, risk perception, and strategies adopted to prevent contracting the infection. We believe the results of our work may be helpful in the development of policies and trainings related to the use of PPE in Italy as well as in other countries.

## Methods

### Study population

We undertook a cross-sectional, self-reported survey, of physicians working in Italy during the response to COVID-19. We disseminated an online survey by the use of two social media groups (via Facebook and WhatsApp) created by physicians engaged in the response. To be part of the groups physicians were required to show a picture of their Italian medical license. This study was found to be exempt by Harvard Longwood Medical’s Institutional Review Board and therefore waived the need for informed consent as this was not found to be human subjects research. The survey was implemented between March 31 and April 5 2020. The population of interest included physicians aged ≥ 21 years with a valid medical license and working in Italy during the emergency (a survey question asked them to report where they worked, including the name of the healthcare unit).

### Survey instrument

The questionnaire was developed through a series of meetings between the researchers and practitioners in charge of infectious control procedures and PPE training activities at the hospital level, the practitioners provided feedback on the content validity and comprehensiveness of the survey instrument before implementation. Questions were designed to inform the development of training and policies in response to the crisis and included questions about the physician’s work experience (years of experience, specialty, experience in COVID-19 units and geographic area of work), and questions related to the use of PPE divided: 1) Access to PPE and strategies to cope with shortage, 2) Information received on the use of PPE, 3) Self-reported ability to perform donning and doffing procedures, and 4) Risk perception of contracting the disease.

### Data analysis

Our analysis examines four dependent variables: 1) access to PPE, 2) use of PPE, 3) self-reported ability to perform donning and doffing procedures and 4) risk perception in the work setting. See Table 1 for related questions and coding. We first performed descriptive statistics for each variable. We then applied ordered logistic regression (ologit command in STATA v16) to the three ordinal variables access to PPE, information on PPE use, and donning and doffing ability and logistic regression (logit command in STATA v16) to the variable risk perception. We tested for bivariate associations between each predictor (years of experience, geographic region, type of position, working in a dedicated COVID-19 unit) and the dependent variables, by means of ordered and logistic regression, using a p-value ≤0.25 as cut-off as inclusion criteria for the multiple regression model [10]. We also tested for collinearity among predictors by Spearman test prior to the completion of the regression analysis. We tested the parallel regression assumption by means of the *Brant* test for the ordered logistic model, which resulted not statistically significant, and as such the *ologit* command was used to run the analysis. Open questions were analyzed to describe strategies that physicians adopted to manage the shortage of PPE and to reduce the risk of infecting their family members. Physicians were first asked to check a box asking if they adopted any strategy and then if they did to specify what strategy. Answers consisted of short sentences, sometimes only few words and as such they were simply coded and described in content and percentages. We used Stata 16 software (Stata, College Station, TX) to analyse the survey data.

**Table 1:** Dependent variables: Survey questions and coding.

Dependent variables	Coding of responses
**Access to PPE:** Do you believe to have adequate access to the PPE necessary for your daily professional activity?	1 = rarely/never
	2 = sometimes
	3 = always
**Use of PPE:** Do you believe you have received adequate information regarding the use of PPE to protect yourself from contracting COVID-19?	1 = rarely/never
	2 = sometimes
	3 = always
**Self-reported ability to perform donning and doffing procedures:** Based on the information received to date do you believe you can correctly don and doff the following pieces of equipment (a list was provided including respirators, masks, gowns, cups and gloves)?	0 = I do not know how to don or doff the piece of equipment
	1 = I am not sure
	2 = I know how to do it
	NB: We combined all responses for each piece of equipment into a scoring system to create a new variable named “*ability to perform donning and doffing procedures”*, We then dichotomized the variable into high ability when the score was⩾75th percentile and less than high when below the 75th percentile)
**Risk perception in the work setting:** What do you believe is your risk of contracting COVID-19 in the work setting in the next 30 days?	Scale 0 = no risk to 100 = high risk subsequently responses were coded as follows:
	1 = low risk (≤ 25th percentile),
	2 = medium risk (26th-75th percentile) and
	3 = high risk (>75th percentile).

## Results

### Characteristics of the study population

Responses were received from 529 physicians, of which 516 were working in Italy, the majority of respondents were in the age category 36–50 (40%), working in the hospital setting as employees of the national healthcare system (59%), and the most frequently reported category for years of experience was 11–20 (30%). Physicians were from all 20 Italian regions and the Republic of San Marino, most respondents were from the Lombardia region (13%), the most impacted by the emergency. Over 40 medical specialities were reported by the respondents, the most frequent of which being Pediatrics (12%), Primary care (7%) and Anesthesiology/Intensive Care (6%) and Cardiology (6%). Details on the sample characteristics are provided in Table 2. Data are available to the public at: https://github.com/esavoia123/COVID19HCWPPE.

**Table 2:** Respondents’ characteristics (n = 516).

**Age**	**N (%)**
18–25	4 (1%)
26–35	89 (17%)
36–50	202 (40%)
51–65	197 (38%)
>65	24 (4%)
**Year of experience in healthcare**	**N (%)**
<5	104 (20%)
5–10	71 (14%)
11–20	155 (30%)
21–30	96 (18%)
>30	90 (17%)
**Region**	**N (%)**
Abruzzo	15 (3%)
Basilicata	2 (0.4%)
Calabria	10 (1.9%)
Campania	39 (7.5%)
Emilia Romagna	45 (9%)
Friuli Venezia Giulia	34 (6%)
Lazio	68 (13%)
Liguria	12 (2.3%)
Lombardia	70 (13.5%)
Marche	10 (2%)
Molise	2 (0.4%)
Piemonte	44 (8.5%)
Puglia	15 (2.9%)
Sardegna	17 (3.3%)
Sicilia	40 (8%)
Toscana	31 (6%)
Trentino Alto Adige	6 (1.2%)
Umbria	18 (3.5%)
Valle d'Aosta	1 (0.2%)
Veneto	36 (6.9%)
Republic of San Marino	1 (0.3%)
Missing	13 (2%)
**Type of employment**	**N (%)**
Employed by the national healthcare system	301 (58%)
Medical Resident	50 (10%)
Adult primary care physician	43 (8%)
Pediatric primary care physician	25 (5%)
Independent contractor	60 (12%)
Ambulatory care physician at the territorial level	11 (2%)
Other	26 (5%)
**Type of unit/clinic**	**N (%)**
Unit dedicated to COVID-19 patients	93 (18%)
Unit non dedicated to COVID-19 patients	330 (64%)
Both type of units (A & B)	50 (10%)
Other	43 (8%)
Specialty*	**N (%)**
Pediatrics	66 (13%)
Primary care	35 (7%)
Anesthesiology—Intensive Care Medicine	29 (6%)
Cardiology	33 (6%)
Psychiatry	27 (5%)
Gynecology -OBGYN	22 (4%)
Radiology	22 (4%)
Emergency Medicine	18 (3%)
Internal Medicine	19 (3%)
Geriatrics	18 (3%)
General Surgery	15 (3%)
Neurology	13 (2%)
Orthopedics	10 (2%)
Gastroenterology	12 (2%)
Preventive Medicine	11 (2%)
Pneumology	8 (1%)
Ophthalmology	8 (1%)
Otolaryngology	7 (1%)
Oncology	7 (1%)
Dentistry	7 (1%)
Occupational Medicine	6 (1%)
Dermatology	5 (1%)
No specialty	43 (8%)
Other	78 (15%)

*Over 40 specialties were reported

### Access to PPE

When asked if they had access to PPE when they needed it, 191 (37%) of the physicians said they rarely or never did, 260 (50%) sometimes and 65 (13%) always did. FFP3 and FFP2 (equivalent to N-99 and N-95 in the USA) were the pieces of equipment most frequently reported as lacking by 59% and 56% of physicians respectively. Other pieces of equipment were also reported as lacking but by a lower percentage of respondents: gown (44%), hair cups (34%), surgical masks (27%), and gloves (16%). Lack of PPE forced 89% of physicians to come up with strategies to cope with the shortage. Such strategies included: using the same N-95 for long shifts (12 hours and beyond), 47% said they were disinfecting the respirator with alcohol, 27% were re-using the same N-95 for multiple shifts and other strategies included adding a surgical mask either under or on top of the N-95, exposing the respirator to “the sun” or to ozone, making masks on their own at home, and buying respirators of unknown certification.

In the bivariate analysis of factors that related to PPE access; working in a COVID-19 unit, in the North or Centre of the country and in a primary care setting were associated with access to PPE, while the variable *years of work experience* was dropped from the final model because of p-value > 0.25. More specifically, in the final ordered multiple logistic model physicians working in COVID-19 units had 3.8 increased odds (OR = 3.8, 95% C.I. 2.5–5.7) of having *access to the PPE they need* at a higher degree of frequency (from never/rarely, sometimes, always) compared to physicians not working in such units. Physicians working in the North and Central area of the country, the most affected regions, also reported higher odds of having adequate access to PPE (OR = 2, 95% C.I. 1.4–3) compared to those working in the South. On the contrary, adult primary care physicians had half the odds (OR = 0.5, 95% C.I. 0.3–0.9) of having access to PPE when they needed it. See Table 3.

**Table 3:** Percent of physicians reporting level of PPE needed compared to recommendations at the time of survey for twelve scenarios.

	NO surgical mask, YES FFP2 (% of the total answers)	NO surgical mask, YES FFP3 (% of the total answers)	NO surgical mask, YES FFP2 OR FFP3 (% of the total answers)	NO surgical mask, NO FFP2 OR FFP3 (% of the total answers)	YES surgical mask, YES FFP2 (% of the total answers)	YES surgical mask, YES FFP3 (% of the total answers)	YES surgical mask, YES FFP2 OR FFP3 (% of the total answers)	YES surgical mask, NO FFP2 OR FFP3 (% of the total answers)	Total answers	Do not know
**Scenario 1: Direct assistance to a COVID-19 positive patient**	**30,9**	**32,0**	**11,7**	**1,2**	**8,7**	**5,6**	**1,0**	**8,9**	**515**	**1 (0.1%)**
**Scenario 2: aerosol generating procedures in a COVID-19 positive patient**	**5,4**	**65,2**	**8,9**	**2,1**	**1,4**	**13,2**	**1,0**	**0,8**	**505**	**11 (2.1%)**
**Scenario 3: Performing oro-pharingeal swab in positive COVID-19 patient**	**20,2**	**48,7**	**7,8**	**2,1**	**3,7**	**10,5**	**1,6**	**3,9**	**507**	**9 (1.7%)**
**Scenario 4: Direct assistance for a non-COVID-19 patient**	**10,1**	**3,7**	**1,7**	**8,7**	**3,9**	**1,4**	**0,2**	**70,3**	**515**	**1 (0.1%)**
**Scenario 5: Transportation of a COVID-19 positive patient by ambulance**	**25,4**	**39,4**	**8,9**	**3,5**	**5,8**	**6,4**	**1,2**	**6,6**	**501**	**15 (2.9%)**
**Scenario 6: Transportation of a suspected COVID-19 patient by ambulance**	**28,2**	**31,7**	**8,3**	**4,3**	**7,8**	**6,0**	**1,4**	**9,5**	**500**	**16 (3.1%)**
**Scenario 7: Transportation of a COVID-19 positive in the hospital**	**29,0**	**33,3**	**8,5**	**4,8**	**6,5**	**7,3**	**1,6**	**9,1**	**498**	**18 (3.4%)**
**Scenario 8: Transportion of a suspected COVID-19 patient in the hospital**	**30,6**	**26,6**	**9,1**	**5,2**	**7,1**	**6,0**	**1,8**	**13,9**	**496**	**20 (3.9%)**
**Scenario 9: Administrative activities**	**3,0**	**0,4**	**0,8**	**25,4**	**0,8**	**0,4**	**0,6**	**70,4**	**505**	**11 (2.1%)**
**Scenario 10: Physical examination of patient with respiratory symptoms**	**35,5**	**21,8**	**7,9**	**4,0**	**7,5**	**4,4**	**2,2**	**19,8**	**511**	**5 (0.9%)**
**Scenario 11: Physical examination of patient without respiratory symptoms**	**7,9**	**3,6**	**1,0**	**25,0**	**13,5**	**6,7**	**2,2**	**42,9**	**510**	**6 (1%)**
**Scenario 12: Preliminary screening without direct contact with the patient / user (triage area)**	**13,5**	**6,5**	**2,0**	**21,2**	**4,6**	**1,6**	**0,4**	**51,0**	**500**	**16 (3.1%)**

### Information about the use of PPE

When physicians were asked how frequently they had *received adequate information regarding the use of PPE to protect themselves from contracting COVID-19*, 132 (26%) reported that they always did, 193 (37%) sometimes, 191 (37%) rarely or never. Approximately half of the physicians reported that the information received to date about the use of PPE was either clear (47%) or complete (54%) and approximately one quarter was unsure about clarity (29%) or completeness (28%), leaving only 25% satisfied with the information they received. When asked if the information received was useful to them, opinions were equally split between three groups: those who found it useful (33%), those who did not (35%), and those who were unsure about its usefulness (31%). As a result of the bivariate analysis years of experience, working in a COVID-19 unit and in a primary care setting were associated to the dependent variable, while geographic area (North, Centre, or South) was dropped from the final ordered logistic multiple model because of p-value > 0.25. In the ordered logistic multiple regression physicians working in a unit dedicated to COVID-19 reported higher odds (OR = 1.8, 95% C.I. 1.2–2.6) of receiving adequate information more frequently compared to physicians not working in such units. On the contrary, adult primary care physicians had 0.5 decreased odds (OR = 0.5, 95% C.I. 0.3–0.9) of receiving such information compared to physicians working in a different setting (hospital or pediatric primary care). See Table 3.

### Ability to perform donning and doffing procedures

**Fig 1:**
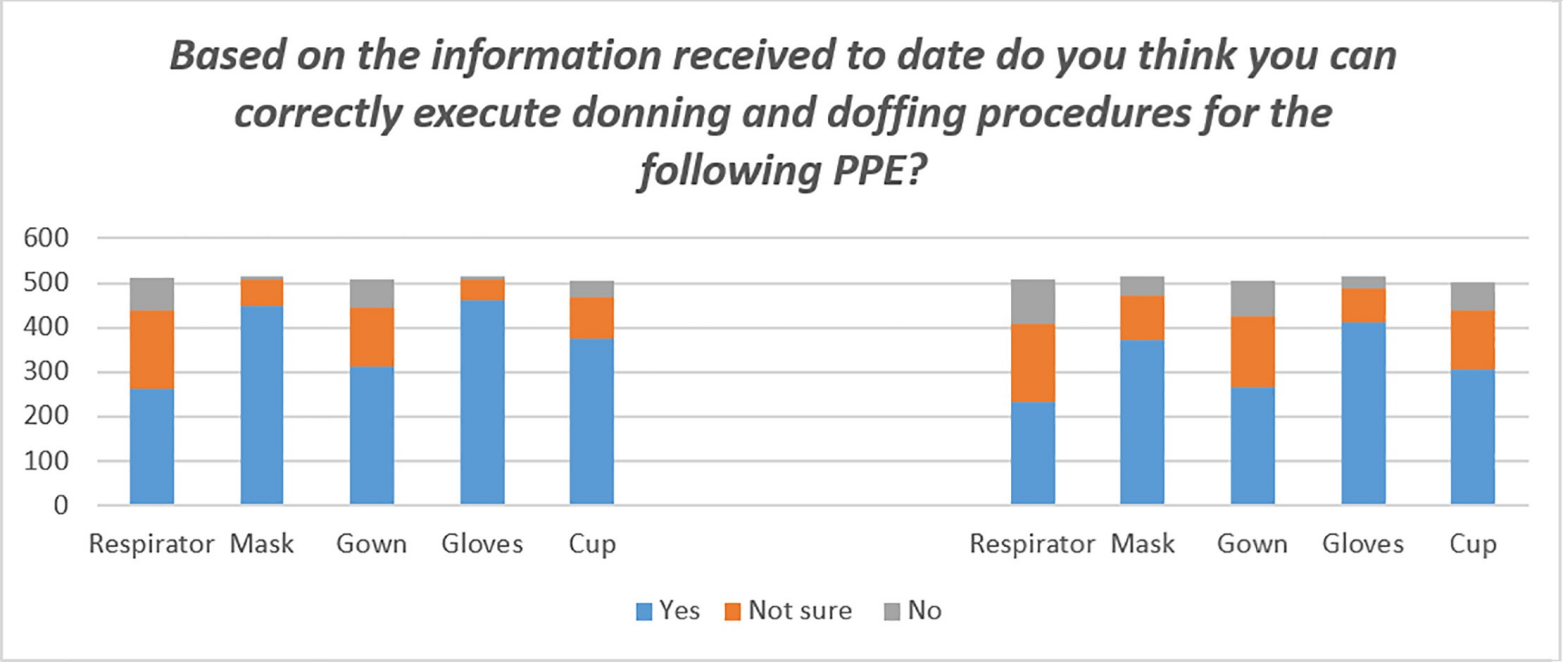
Ability to perform donning and doffing procedures by piece of PPE.

When asked if they believed they could correctly execute donning and doffing procedures for specific pieces of PPE, respondents felt mostly unprepared for putting the respirators and gowns on (14% and 13% respectively) or unsure if they were doing it correctly (34% and 25%). In regards to doffing, once again, taking off the respirator and the gown were the procedures they did not know how to do correctly (19% and 16%) or were unsure about (34% and 32%). See Fig 1. In the multiple logistic regression model years of experience, working in a COVID-19 unit, and frequency of information received about the use of PPE were associated with better donning and doffing performance. Results show that for each increase in the category of years of experience physicians reported 1.3 greater odds of being able to perform the procedures [OR = 1.3, 95% C.I. 1.1–1.5], those working in COVID-19 units have twice the odds of reporting being able to correctly perform the procedures [OR = 2.3, 95% C.I. 1.5–3.5], and finally those who reported to have received adequate information during the epidemic also reported greater odds of being able to perform the procedures [OR = 2.4, 95% C.I. 1.9–3.2]. See Table 3.

### Accuracy of PPE knowledge based on current guidance

As part of the survey we also presented the physicians with 12 scenarios of activities that would require the use of different types of PPE and asked them, based on their knowledge, what was the most appropriate piece of equipment for each activity. Scenarios are presented according to an implicit scale of frequency of actions described: being scenarios 1–3 the ones representing the most common and most risky activities in attending patients; scenarios 4 concerned the no COVID patients; scenarios 5–8 were tackling the issue of patients transportation; scenario 9 described administrative tasks; scenarios 10 and 11 regarded physical examination of patient with or without respiratory symptoms and scenario 12 was about the first contact with patients in a triage area. Overall respondents assigned to each activity a level of protection higher compared to what was recommended by national and regional guiding documents published at the time of the survey, in Table 3 [10]. In Scenario 1, we observed that one fourth of respondents (24,2%) considered surgical mask the correct PPE for personal and patients protection during direct assistance; but only 8,9% considered surgical masks alone enough in protecting, while 74,6% considered necessary a FFP. Scenarios 2 and 3 investigated level of PPE needed for highly risky medical practices concerning COVID-19: aerosol generation and swab performing. Answers given showed that 65,2% would use a FFP3 alone for aerosol generation procedures, and 13,2% will use surgical mask and/or FFP3. While for swab performing, almost half of respondents 48,7% said they would use a FFP3 instead of a FFP2 (20%) which was the correct PPE indicated by GL for this procedure. Surprisingly 70,3% of respondents said that they would use only a surgical mask when attending a non Covid patient. Scenarios 5–8: for transport by ambulance of a confirmed Covid19 patient, only 6,6% considered enough the surgical mask alone, which was the recommended PPE by guidelines. Instead, more than 70% said they would use a FFP2 or FFP3 rather than a surgical mask, against a 13,4% that would use a surgical mask combined with a FFP2 or FFP3. For transport by ambulance of suspected cases (Scenario 6) almost 70% of respondents said they would use a FFP, instead of a surgical mask as indicated by ISS guidelines. Roughly the same percentage of respondents, respectively 70,8% and 66,3%, answered they would use a FFP for transport of confirmed and suspected patients inside the hospital. Scenario 9 portrays the common idea that for administrative tasks, health workers should use a PPE: 70,4% believed that for activities not directly involving contact with patients but within a hospital or health environment, it was appropriated to use a surgical mask. The remaining one third reported there was no need for any personal protection (25,4%). In Scenario 10, we asked physicians what they would wear in case of physical examination of patients with respiratory symptoms (cough, coryza, running nose, dyspnea): the answer was that 65,2% would use a FFP rather than a surgical mask, 19,8% would use a surgical mask without a FFP, and 14% would use both surgical masks and FFP. Only 4% would not use anything. Scenario 11: while performing physical examination of patients without respiratory symptoms, 42,9% said they were confident they did not need to wear any FFP, but the surgical mask was enough. While 25% thought they did not need any protective device at all. However 13% felt more protected by using a surgical mask and a FFP2. The last Scenario, number 12, depicting a situation of triage activity, half of physicians (51,0%) responded they would use a surgical mask, not a FFP.

### Risk perception

When physicians were asked to rate their perceived risk, on a scale from 0 to 100, of contracting the infection in the healthcare setting, they attributed a mean value of 56 (SD = 22) to such risk, the same perception of risk for their life outside the work environment was much lower 25 (SD = 19) T test p<0.001. Though physicians perceived the risk of contracting COVID-19 outside of work to be less, they were still concerned about infecting their family members upon return home from work. The majority of the respondents (72%) reported to have taken precautions at home to keep their family safe; the most cited precaution was to isolate from the rest of the family (45%), other examples consisted of removing and washing their clothes upon arrival at home and wearing a surgical mask, not sharing utensils and keeping a physical distance from family members. Interestingly, none reported to check their body temperature at home. In an attempt to strengthen their immune system and prevent the infection, despite lack of evidence on the matter 37% of respondents reported to have taken vitamins (mainly C and D) and (1%) self-administered hydroxycloroquine. In the multivariable ordered logistic model of factors related to perceived risk at work, those working in COVID-19 units showed 3 times greater odds of reporting a higher level of risk perception compared to those not working in such units [OR = 3, 95% C.I. 2–4.6]. On the contrary those with access to PPE showed lower odds of reporting higher risk perception [OR = 0.6, 95% C.I 0.4–0.7] compared to those who do not to have access to the equipment, and so do those who report to have received adequate information on how to use the PPE [OR = 0.6, 95% C.I. 0.4–0.7]. Interestingly, the ability to conduct donning or doffing procedures was not associated with risk perception. See Table 4.

**Table 4:** Predictors of physicians’ access to PPE, information about the use of PPE, donning and doffing performance, and risk perception at work (n = 516).

Independent variables	Multiple regression models—Odds ratios and 95% confidence limits			
	Access to PPE*	Information received about the use of PPE*	Ability to perform donning and doffing procedures**	Risk perception of contracting COVID-19 at work*
Years of work experience (<5;5–10;11–20;21–30;>30)	Excluded after bivariate analysis	1.1 [1–1.3]	1.3 [1.1–1.5]	Excluded after bivariate analysis
Working in a COVID-19 unit (Yes = 1; No = 0)	3.8 [2.5–5.7]	1.8 [1.2–2.5]	2.3 [1.5–3.5]	3 [1.4–2.9]
Region (North/Central = 1; South = 0)	2 [1.4–3]	Excluded after bivariate analysis	1.5 [1–2.3]	Excluded after bivariate analysis
Working as an adult primary care physician (Yes = 1; No = 0)	0.5 [0.3–1]	0.6 [0.3–1]	0.5 [0.2–1]	Excluded after bivariate analysis
Receiving adequate information about the use of PPE (Never/Rarely = 1;2 = Sometimes;3 = Always)	Excluded after bivariate analysis	Excluded after bivariate analysis	2.4 [1.9–3.2]	0.5 [0.4–0.7]
Adequate access to PPE (Never/Rarely = 1;2 = Sometimes;3 = Always)	Not included	Not included	Not included	0.6 [0.5–0.8]

*ordered logistic regression

** logistic regression

N/A = excluded after bivariate analysis

## Discussion

Our results present some of the first evidence on how Italian physicians experienced lack of PPE, and what factors influenced their understanding of PPE procedures and use. While our results are not generalizable to the all population of Italian physicians, and certainly derived from a group of physicians with high level of interest in COVID-19, group differences within our sample rather than general group estimates by extrapolation, can be useful to understand predictors of behaviors and specific challenges in access and use of PPE. The majority of those surveyed reported not to have access to PPE every time they need it and at least one third of them reported not having received adequate information on the use of the equipment, nor were they consistently comfortable with donning and doffing procedures, in particular when using respirators and wearing gowns. Working in a COVID unit made a difference in multivariate analysis of both having access to PPE, adequate information on their use, feeling comfortable with donning and doffing procedures, and perceived risk. This likely reflects training efforts focused on educating this subset of the workforce, those actually at the highest risk of contracting COVID-19 based on occupational risk. However, given the difficulties of creating 100% COVID-19 free clinics as many patients may present to a clinic in a pauci-symptomatic status, the current variation in access and knowledge about PPE use, may put at a disproportionate risk those working outside COVID-19 units. More specifically, our results indicate how primary care physicians may have been neglected from informational initiatives posing them at high risk of contracting the infection. With the advancement of testing and treatment options in the months ahead more and more COVID-19 patients will be diagnosed and cured outside the hospital setting. Therefore, additional attention is needed to provide PPE and PPE training for this group of providers and all those working outside COVID-19 units.

Interestingly, respondents consistently overestimated the level of PPE needed to interact with a non-symptomatic patient; reflecting they either had inadequate understanding of current guidance or regardless of the guidance they were fearful of becoming infected themselves and/or infecting the patient when a diagnosis was not confirmed. As responses to scenarios 5–8 reflect, the fact that the risk of handling patients (transmission through contact), regardless of the physical space and environment, was perceived as higher than being in a closer contact with them (droplets), or it can be just a matter of not being directly involved in the transport of patients. Further, results reflect physicians' concerns were highest when handling respiratory symptoms, a logical response given at the time of the survey asymptomatic transmission and the range of symptoms related to COVID-19 were not wholly understood. The ongoing changes to PPE guidance provided by international, national and regional public health agencies, in particular in regards to the use of respirators, likely made it more challenging to make sense of which equipment to use, something that likely has continued as the constellation of symptoms that COVID-19 presents have continued to evolve. Standards of use shifted in mid-March, a couple of weeks prior to our survey, as a result of PPE shortages and lack of logistics planning within hospitals [11]. Limited training as well as pre-existing professional norms that lacked a culture of PPE use may have been factors that shaped challenges in developing adequate training and information material. We suggest that future efforts should be made to include PPE training in the medical curriculum so that in times of crisis physicians can better adapt to their use and differences in knowledge and practices would be less evident across categories. Methods for just-in-time training including the use of video trainings may be one mechanism to improve donning and doffing procedures [12]. In times of crisis, an overuse and gauging of PPE by concerned physicians may cause as much harm as lack of supplies.

We found PPE perceptions and use were also tied to perceived risk of contracting the infection in the work environment. Overall, risk perception was high, but both adequate access and PPE training decreased such perception. Of concern, is also the fact that many physicians took actions in their personal lives to protect their families, limiting physical interactions and in some cases renting separate apartments. In the long term, these actions will certainly affect their emotional well-being.

## Conclusion

Results from this rapid survey indicate that while ramping up supplies on PPE for healthcare workers is a necessity, adequate training and clear instructions are just as important. To the extent possible instructions need to be consistent overtime and across regions, include recommendations not only on the overall safety of the workers in the healthcare setting but also on strategies to maintain their overall physical and emotional health and the health of their loved ones.
